# IMPACT OF SOCIAL ISOLATION ON OLDER ADULTS IN NURSING HOMES DURING THE PANDEMIC LOCKDOWN: INFORMING POLICY

**DOI:** 10.1093/geroni/igac059.033

**Published:** 2022-12-20

**Authors:** Rebecca Koszalinski, Diana Sturdevant, Patsy Smith, Julie Myers, Debara Yellseagle, Brenda Olmos, Molly Netter

**Affiliations:** OUHSC, Oklahoma City, Oklahoma, United States; University of Oklahoma HSC, Oklahoma City, Oklahoma, United States; University of Oklahoma HSC, Oklahoma City, Oklahoma, United States; University of Oklahoma Health Sciences Center, Oklahoma City, Oklahoma, United States; University of Oklahoma HSC, Oklahoma City, Oklahoma, United States; University of Oklahoma HSC, Oklahoma City, Oklahoma, United States; University of Oklahoma HSC, Oklahoma City, Oklahoma, United States

## Abstract

**Purpose:**

Describe outcomes of an equity-centered community design (ECCD) project examining the impact of COVID-19 restrictions and social isolation on nursing home (NH) owners, administrators, community leaders, clergy, and urban homeless shelter staff. •

**Methods:**

3-phase, ECCD project. Key informants (KI) were recruited through snowball sampling if experienced in caring for older adults (OA) in NH, in the community, or were engaged in spiritual care of OA.•

**Results:**

Perceptions of fear, anger, frustration, sadness, and concern. Categories of Lost Communication and Socialization Strategies, Lost Freedom, Residents’ Dying Alone and Inability to Pause to Grieve, Helplessness and Hopelessness, Inability to Take Time to Grieve, and Behavior Changes. KI offered New Ways of Communicating, and interventions and solutions were generated in Phase III.• Implications. Understanding of Stakeholder experiences and perceptions is important to achieving inclusive, equity-centered solutions. Future policy decisions must consider what matters most to those most directly impacted by those decisions.

